# Vanishing Bile Duct Syndrome as an Uncommon Hepatic Paraneoplastic Syndrome in Hodgkin’s Lymphoma

**DOI:** 10.1155/crhe/4166844

**Published:** 2026-02-10

**Authors:** Natasha Salman, Diana Sittirat Petchpailin, Amanda Seemann, Apurva Modi, Rohan Gupta, Shovendra Gautam

**Affiliations:** ^1^ Internal Medicine Residency Department, Baylor Scott & White All Saints Medical Center Fort Worth, Fort Worth, Texas, USA

**Keywords:** bile duct, biliary cirrhosis, cholestasis, Hodgkin’s lymphoma, liver biopsy, liver cirrhosis, paraneoplastic syndrome, vanishing bile duct syndrome

## Abstract

Vanishing bile duct syndrome (VBDS) is a rare form of liver injury caused by ischemia, drug reactions, autoimmune diseases, infections, or malignancy. VBDS involves the progressive disappearance of intrahepatic bile ducts, causing cholestasis and biliary cirrhosis, with high mortality if untreated. VBDS can also present as a paraneoplastic syndrome in Hodgkin’s lymphoma (HL). A 53‐year‐old female presented with jaundice, pruritus, diarrhea, and elevated liver function tests (LFTs). A liver biopsy demonstrated cholestasis with mild ductopenia, and imaging revealed enlarged para‐aortic lymph nodes. A bone marrow biopsy confirmed HL, and chemotherapy normalized symptoms and LFTs.

## 1. Introduction

Vanishing bile duct syndrome (VBDS) is a rare type of liver injury characterized by chronic cholestasis and progressive loss of intrahepatic bile ducts. VBDS develops following episodes of cholestatic hepatitis with immunologic features. Risk factors for VBDS include ischemia, neoplastic processes, graft‐versus‐host disease, autoimmune disorders, and infectious diseases [1]. VBDS has been reported in approximately 40 cases of Hodgkin’s lymphoma (HL), arising from an imbalance between biliary epithelial cell apoptosis and regeneration, with apoptosis predominating due to increased B‐cell lymphoma‐2‐associated X‐protein (BAX) and tumor necrosis factor‐alpha (TNF‐α) [2–4].

## 2. Case Presentation

A 53‐year‐old female with no preexisting liver disease presented with jaundice, pruritus, vomiting, and diarrhea after undergoing abdominoplasty and suction‐assisted lipectomy complicated by a postoperative infection treated with cephalexin, acetaminophen, and gabapentin. Labs revealed elevated alkaline phosphatase (ALP) (564), alanine aminotransferase (ALT) (271), aspartate aminotransferase (AST) (287), and total bilirubin (6.4). Preoperative labs showed an ALP of 150. Abdominal imaging demonstrated multiple enlarged periaortic and periportal lymph nodes, considered reactive lymphadenopathy. Magnetic resonance cholangiopancreatography (MRCP) showed no bile duct dilation or malignant strictures. A liver biopsy showed mild lobular inflammation and cholangitis with cholestasis, without fibrosis. A diagnosis of drug‐induced liver injury (DILI) secondary to cephalexin was favored, and the patient was started on prednisone.

Despite discontinuing cephalexin and receiving corticosteroids, the patient’s liver function tests (LFTs) worsened. Serologic testing revealed elevated Epstein–Barr virus (EBV) immunoglobulin G (IgG), indicating prior exposure, but was negative for active EBV infection, hepatitis A virus (HAV), hepatitis B virus (HBV), hepatitis C virus (HCV), cytomegalovirus (CMV), herpes simplex virus (HSV), and human immunodeficiency virus (HIV). Follow‐up MRCP demonstrated nonspecific left periaortic adenopathy, and CT imaging revealed enlarged left para‐aortic lymph nodes. A liver biopsy showed cholestasis and mild ductopenia. A CT‐guided lymph node biopsy demonstrated sclerosis without malignancy, and flow cytometry was unremarkable.

Three months later, the patient had persistently elevated ALP (> 1000) and bilirubin (> 12). Imaging revealed T2 hyperintense, enhancing lesions in the vertebrae, pelvis, and sacrum, along with widespread lymphadenopathy suggesting metastatic lymphoma. A bone marrow biopsy confirmed Stage IV BHL. An excisional lymph node biopsy confirmed nodular sclerosis HL.

The patient began pembrolizumab but, due to lack of improvement, was transitioned to hyperfractionated cyclophosphamide with dexamethasone, followed by R‐GemOx (gemcitabine–oxaliplatin + rituximab). After five cycles of adriamycin, bleomycin sulfate, vinblastine sulfate, and dacarbazine (ABVD), clinical remission was achieved, and LFTs improved significantly: AST decreased from 270 to 114, ALT from 105 to 61, ALP from 1943 to 507, and total bilirubin from 9.6 to 4.2. Given her clinical progression, lymphoma diagnosis, and liver biopsy findings of ductopenia, VBDS was diagnosed.

**FIGURE 1:**
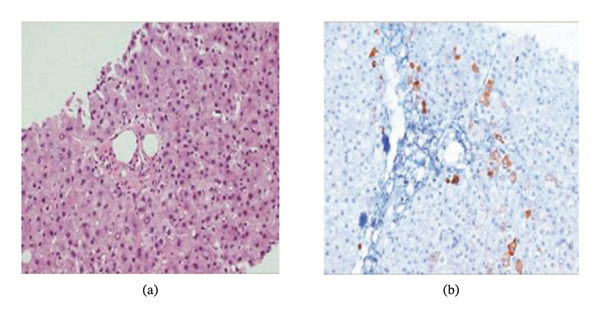
Vanishing bile duct syndrome in a patient with Hodgkin’s lymphoma [3]. These are liver biopsy specimens from patients with bile duct loss. (a) A portal tract with almost no bile ducts present. There is also necrosis and inflammatory infiltrates such as plasma cells present around the bile ducts. (b) Immunohistochemical staining with CK7 reveals ductal proliferation in response to chronic bile duct loss, a process that occurs as the liver attempts to regenerate bile duct structures to replace the lost or injured bile ducts. The hepatocytes (arrowhead) indicate cholangial metaplasia, the transformation of nonbile duct cells into cells resembling bile duct cells. This is an adaptive response to bile duct damage.

The patient underwent autologous stem cell transplantation (ASCT) with single‐agent melphalan 200. At her most recent follow‐up, LFTs had normalized with AST 20, ALT < 5, ALP 100, and bilirubin 0.5 (Figure 1).

## 3. Discussion

VBDS is a rare liver injury caused by drugs, autoimmune diseases, infections, or malignancies. Medications such as sertraline, lamotrigine, trimethoprim–sulfamethoxazole (TMP–SMX), ibuprofen, levofloxacin, and more are commonly implicated, with patients often fully recovering after medication cessation [5]. Infections such as HIV, CMV, and EBV are known to trigger VBDS, along with both HL and non‐HL and conditions such as primary biliary cholangitis, primary sclerosing cholangitis, sarcoidosis, and chronic graft‐versus‐host disease [6]. VBDS causes loss of bile ducts, leading to cholestasis and ductopenia, accounting for 0.5% of small duct biliary disease [7]. Ductopenia is defined as the absence of interlobular bile ducts in over 50% of small portal tracts on liver biopsy [5].

VBDS presentation varies, with symptoms such as weight loss, pruritus, and jaundice. The extent of bile duct damage can range from irreversible duct loss with extensive ductopenia, fibrosis, or cirrhosis, to clinical recovery within months to years [8].

VBDS can occur without known risk factors, as shown in a 2022 case of a woman who presented with fever, jaundice, and elevated liver enzymes. A liver biopsy revealed significant bile duct loss, and treatment with ursodeoxycholic acid and prednisone normalized her liver enzymes, with stable liver function on follow‐up [5]. Similarly, a 25‐year‐old woman developed VBDS after a weight loss regimen using various medications and herbal remedies. She presented with jaundice, pruritus, and elevated liver enzymes. After discontinuing hepatotoxic substances and starting ursodeoxycholic acid, her symptoms improved, but hyperbilirubinemia recurred. A liver biopsy revealed intrahepatic bile duct loss. Imaging revealed splenomegaly, and a lymph node biopsy confirmed nodular sclerosis HL. She was treated with prednisone and chemotherapy, resulting in clinical improvement [9].

Liver biopsy is essential for the diagnosis of VBDS, with ductopenia defined as the absence of interlobular bile ducts in at least 50% of small portal tracts. It is important to distinguish VBDS from idiopathic cholestasis where ductopenia is absent and liver function improves with therapy [8]. There is no standardized treatment for VBDS. In medication‐induced VBDS, discontinuing the offending agent often resolves symptoms. Corticosteroids are effective in severe cases of VBDS, while ursodeoxycholic acid is frequently used to promote cholangiocyte survival by inhibiting intrinsic apoptosis [5]. It is important to note that a component of DILI cannot be entirely excluded in this context. Drug‐induced VBDS may persist despite withdrawal of the offending agent, and hepatic injury can evolve into a chronic process lasting longer than 6 months after drug cessation. Although alternative etiologies such as paraneoplastic VBDS associated with HL are strongly supported, a medication‐related contribution cannot be definitively ruled out and remains an important consideration in the differential diagnosis.

Hepatic involvement occurs in up to 50% of HL patients, but cholestatic liver injury is seen in less than 4% [6, 10]. VBDS as a paraneoplastic manifestation of HL was first described in 1993, with initial cases progressing to liver failure. Hepatic injury can serve as an early indicator of HL, as seen in cases where patients initially presented with elevated LFTs and subsequent liver biopsy revealed cholestatic hepatitis prior to the diagnosis of HL. Although not all patients with VBDS develop liver failure, its presence in HL is associated with high mortality.

The pathogenesis of VBDS in HL is unclear but likely involves immune‐mediated mechanisms such as cytokine release from lymphoma cells causing ductopenia, or T‐cell–induced apoptosis of biliary epithelial cells [6]. Increased expression of major histocompatibility complex (MHC) in response to HL cytokines suggests an immune‐mediated process driven by the production of autoantibodies by HL or T‐cell toxicity against the biliary epithelium.

In HL patients with VBDS, the goal is remission; however, chemoradiation can exacerbate liver dysfunction, sometimes necessitating liver transplantation. A multidisciplinary approach combining lymphoma treatment with liver transplant evaluation may improve survival [6]. Emerging therapies targeting immune mechanisms underlying VBDS, such as rituximab, have shown promise in improving outcomes [11].

**Nomenclature**
ALP: Alkaline phosphatase
ALT: Alanine aminotransferase
AST: Aspartate aminotransferase
CPI: Checkpoint inhibitor therapy
CMV: Cytomegalovirus
CT: Computed tomography
DILI: Drug‐induced liver injury
EBV: Epstein–Barr virus
HAV: Hepatitis A virus
HBV: Hepatitis B virus
HCV: Hepatitis C virus
HL: Hodgkin’s lymphoma
HSV: Herpes simplex virus
HIV: Human immunodeficiency virus
IgG: Immunoglobulin G
INR: Internalized normalized ratio
TMP–SMX: Trimethoprim–sulfamethoxazole
LFTs: Liver function tests
MRCP: Magnetic resonance cholangiopancreatography
VBDS: Vanishing bile duct syndrome

## Funding

No funding was received for this manuscript.

## Disclosure

Portions of this case report were previously presented as an abstract at the SOHO 2024 Twelfth Annual Meeting and published in *Clinical Lymphoma, Myeloma and Leukemia*, Volume 24, Supplement 1, September 2024.

## Consent

Consent was obtained or waived by all participants in this study.

## Conflicts of Interest

The authors declare no conflicts of interest.
